# Prevention of diabetes-promoted colorectal cancer by (n-3) polyunsaturated fatty acids and (n-3) PUFA mimetic

**DOI:** 10.18632/oncotarget.2453

**Published:** 2014-09-08

**Authors:** Anna Algamas-Dimantov, Einav Yehuda-Shnaidman, Rachel Hertz, Irena Peri, Jacob Bar-Tana, Betty Schwartz

**Affiliations:** ^1^ Institute of Biochemistry, Food Science and Nutrition, The Robert H. Smith Faculty of Agriculture, Food and Environment, The Hebrew University of Jerusalem, Israel; ^2^ Department of Human Nutrition and Metabolism, Hebrew University Medical School, Jerusalem, Israel

**Keywords:** Colorectal cancer, diabetes, obesity

## Abstract

The global obesity / diabetes epidemic has resulted in robust increase in the incidence of colorectal cancer (CRC). Epidemiological, animal and human studies have indicated efficacy of (n-3) PUFA in chemoprevention of sporadic and genetic-driven CRC. However, diabetes-promoted CRC presents a treatment challenge that surpasses that of sporadic CRC. This report analyzes the efficacy of (n-3) PUFA generated by the fat-1 transgene that encodes an (n-6) to (n-3) PUFA desaturase, and of synthetic (n-3) PUFA mimetic (MEDICA analog), to suppress CRC development in carcinogen-induced diabetes-promoted animal model. Carcinogen-induced CRC is shown here to be promoted by the diabetes context, in terms of increased aberrant crypt foci (ACF) load, cell proliferation and epithelial dedifferentiation, being accompanied by increase in the expression of HNF4α, β-catenin, and β-catenin-responsive genes. Incorporating the fat-1 transgene in the diabetes context, or oral MEDICA treatment, resulted in ameliorating the diabetic phenotype and in abrogating CRC, with decrease in ACF load, cell proliferation and the expression of HNF-4α, β-catenin, and β-catenin-responsive genes. The specificity of (n-3) PUFA in abrogating CRC development, as contrasted with enhancing CRC by (n-6) PUFA, was similarly verified in CRC cell lines. These findings may indicate prospective therapeutic potential of (n-3) PUFA or MEDICA in the management of CRC, in particular diabetes-promoted CRC.

## INTRODUCTION

Colorectal cancer (CRC) is the second most common cause of cancer in women and the third most common in men, being the fourth cause of cancer death. The global obesity / diabetes epidemic has resulted in robust increase in the incidence of CRC [1], with 7% increase in CRC risk for every 2.4 unit increase in body mass index (BMI) [2]. Type 2 diabetes (T2D) and insulin treatment may further result in doubling the risk for CRC [3]. CRC initiation and promotion by diabetes is due to diabetes-induced increase in growth factors (e.g., insulin, IGF-1) and inflammatory adipo/cytokines (e.g., TNFα, IL-6) (reviewed in [4]).

Most sporadic CRC is due to lifestyle and increasing age with only a minority being driven by underlying genetic disorders. Epidemiological studies have indicated variable decrease in sporadic CRC risk (RR 0.67-0.88) upon (n-3) PUFA consumption, with increase in colorectal adenoma risk (RR 1.68) by (n-6) PUFA consumption [5]. These studies were further corroborated by animal studies whereby (n-3) PUFA, administered during the pre- or post-initiation phase, prevented azoxymethane (AOM)- or dimethylhydrazine (DMH)-induced CRC compared with (n-6) PUFA (reviewed in [6] ). Similar efficacy of (n-3) PUFA has been reported in Apc^Min^ mouse models [7]. CRC prevention was accounted for by (n-3) PUFA activity per se, rather than reduction in (n-6) PUFA intake [7]. (N-3) PUFA was further shown to abrogate the development of CRC tumors xenografted in immunocompromised mice [8], and to reduce the number and size of liver CRC metastases [9]. That is in contrast to increase in number and size of liver CRC metastases by supplementation of (n-6) PUFA (reviewed in [10]). (N-3) PUFA efficacy in animal models was further confirmed by reduction in mucosal epithelial cell proliferation index in patients with sporadic colorectal adenoma [11]. Most importantly, rectal polyp multiplicity and size were significantly reduced by (n-3) PUFA supplementation in patients with Familial adenomatous polyposis (FAP) [12]. (N-3) PUFA efficacy in ameliorating the course of CRC development has been ascribed to modulation of COX activity, disruption of cell surface lipid rafts, increased oxidative stress, and modulation of the activity of specific transcription factors (e.g. HNF-4α, PPARγ/RXR, SREBP1c) (reviewed in [10]).

Our previous study [13] has indicated that colonic ontogenesis in obese diabetic db/db mice displays CRC-like characteristics, namely, proliferation and de-differentiation of epithelial colonocytes and goblet cells, driven by increase in the expression and transcriptional activities of colonic hepatocyte nuclear factor 4α (HNF-4α) and β-catenin/Tcf. CRC-like ontogenesis in db/db mice, and the expression of β-catenin and HNF-4α-responsive genes, reverted all to the wild-type phenotype, by crossing the db/db mice with the fat-1 transgene that encodes an (n-6) to (n-3) PUFA desaturase [14], resulting in colonic (n-3) PUFA enrichment. (N-3) PUFA effects were ascribed to suppression of HNF-4α transcriptional activity by HNF4α-bound (n-3) PUFA at the expense of decreasing HNF-4α-bound (n-6) PUFA [13]. These studies prompted our interest in pursuing the efficacy of (n-3) PUFA in ameliorating carcinogen-induced CRC in the obese / diabetes context.

Substituted long-chain dicarboxylic acids (MEDICA analogs) mimic the activity of (n-3) PUFA in suppressing the transcriptional activity of HNF4α, while avoiding β-oxidation or esterification into lipids [15]. MEDICA analogues are mostly excreted in bile as respective glucuronides. MEDICA analogs have been further reported to ameliorate T2D in animal models [16-19]. The combined efficacy of MEDICA analogs in treating diabetes, while mimicking (n-3) PUFA activity in suppressing HNF-4α transcriptional activity [15, 20], prompted our interest in probing their efficacy in suppressing carcinogen-induced diabetes-promoted CRC.

## RESULTS

### Strain Phenotypes

Colorectal cancer was induced in the C57BL, fat-1, BKS. Cg db/db, and BKS.Cg db/db crossed with fat-1 (fxB) mouse strains by weekly DMH injections for 4 weeks. Blood glucose, insulin and leptin levels were measured in the four strains 19 weeks following first DMH-administration, and are presented in Table 1. In line with the db/db phenotype, body weight, leptin, insulin and glucose levels were significantly increased in BKS.Cg mice, being partially reverted to control values in the fxB cross ( Table 1), while maintaining food consumption (not shown). The phenotypes of the four strains are similar to those previously reported in non-DMH-treated respective mice [13], implying that DMH treatment didn’t affect the inherent T2D phenotype of the concerned mouse strains.

**Table 1:** Phenotypes of the treated mice strains

Mouse Strains	Body weight (gr)	Leptin (ng/ml)	Insulin (μU/ml)	Glucose (mg/dl)
C57BL/6	25 ± 2.3	6 ± 0.5	63 ± 6.9	72 ± 7.8
fat-1	28 ± 2.7	5 ± 0.6	61 ± 6.5	78 ± 8.5
BKS.Cg	48 ± 5.6^*^	39 ± 4.1^*^	126 ± 14^*^	205 ± 22^*^
BKS. Cg / fat-1 (fXB)	29 ± 3.2^#^	18 ± 2.5^*^^#^	95 ± 9.8^*^^#^	109 ± 11^*^^#^
BKS.Cg / MEDICA	29 ± 3.1^#^	12.2 ± 5.7^*^^#^	85.1 ± 1.5^*^^#^	101.75 ± 7.2^*^^#^

Body weight, fasting blood leptin, insulin and glucose levels were determined 19 weeks following first DMH administration as described in Methods. Mean ± S.E. (n = 6-10 for each strain).

* Significant as compared with C57BL (P<0.001);

# Significant as compared with BKS.Cg (P<0.01).

### ACF scoring

Aberrant crypt foci (ACF) precede colon cancer, and ACF multiplicity may therefore indicate progression of DMH-induced CRC [21]. Representative photomicrographs of ACF multiplicity are presented in Supp Fig 1. ACF multiplicities of the four mouse groups (C57BL, fat-1, BKS.Cg db/db, fxB) were scored at two time points, namely, 11 (cancer initiation) and 19 (cancer progression) weeks from first DMH administration (Table 2). Overall ACF multiplicity is presented in the last row of Table 2, and may reflect the aberrant foci load of respective mouse groups. As shown in Table 2, the aberrant foci load of BKS.Cg db/db mice was significantly higher as compared with C57Bl control mice at the two respective time points, implying increased CRC progression in diabetic mice. In contrast, the aberrant foci load as well as microadenoma (ACF grade more than 6) of fat-1 mice was robustly decreased compared to C57BL control mice at the two respective time points, implying resistance to CRC development by (n-3) PUFA. Most importantly, introducing the fat-1 gene into the db/db context resulted in reverting the aberrant foci load to the fat-1 score, both in terms of the overall load as well as the microadenoma prevalence, implying resistance to DMH-induced diabetes-promoted CRC by (n-3) PUFA.

**Table 2:** ACF scoring

ACF No./2cm colon	C57BL/6 11w	C57BL/6 19w	fat-1 11w	fat-1 19w	BKS.Cg 11w	BKS.Cg 19w	fxB 11w	fxB 19w
1	4 ± 0.4	6±2.0	4 ± 0.5^#^	6±2.1^#^	10 ± 5.0^*^	14± 0.5^*^	8 ± 0.7^#^ ^*^	9±2.0
2	0	10±2.0	2± 1. 7 ^#*^	5±0.9^#^ ^*^	12 ±1.0 ^*^	15±2.5	0^#^	6±1.9^#^
3	0	3±2.0	0	4±1.1	5± 2.0^*^	6±0.1	2± 0.9^#^ ^*^	2±0.5^#^
4	6±2.7	2±0.6	0	5±2.0	7± 3.5	4±2.0	4±1.4^#^	3±1.20
5	4± 1.5	0	0	2±1.5^#^	7±0.7^*^	9±0.5^*^	1±0.0^#^ ^*^	1±0.5^#^
>6	4±0.9	43±7.4	0	0^#^ ^*^	8±0.5^*^	52±4.0 ^*^	1±0.3^#^ ^*^	5±1.3^#^ ^*^
Overall aberrant foci load	72	301	8	58	160	435	41	76

ACF of respective mouse strains were scored on 11 and 19 weeks following the first DMH injection as described in Methods. Overall aberrant foci load summarizes the total foci in the different mice strains 11 and 19 weeks following first DMH administration; Mean ± S.E. (n = 6 mice for each strain).

* - Significant as compared with C57BL (P < 0.05).

# - Significant as compared with BKS.Cg (P < 0.05).

### Colonic proliferation and differentiation

**Figure 1:**
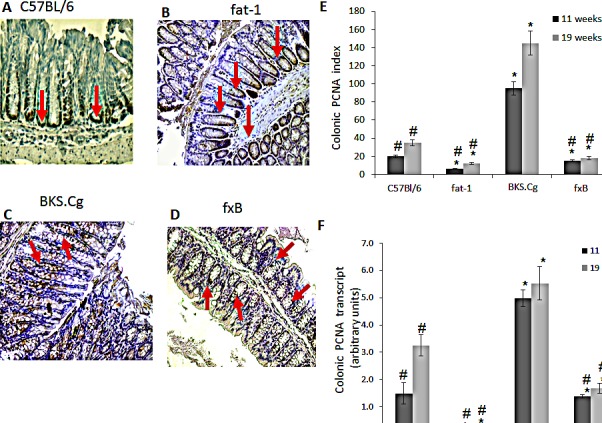
Colonic PCNA expression A-D. Representative colonic PCNA immunohistograms (magnification x 200) of C57BL/6 (A), fat-1 (B), BKS.Cg (C) and fXB (D) mice following 11 weeks from first DMH injection. Arrows: PCNA positive nuclear staining. E. Colonic PCNA index 11 and 19 weeks from first DMH injection. Mean ± S.E. (n = 6 for each strain and time period). *Significant as compared with respective C57BL/6 control mice (*P* < 0.01). ^#^Significant as compared with respective BKS.Cg mice (P <0.02). F. Colonic PCNA transcript levels following 11 and 19 weeks from first DMH administration. Mean ± S.E. (n = 10 for each strain and time period). *Significant as compared with respective C57BL/6 control mice (*P* < 0.001). ^#^Significant as compared with respective BKS.Cg mice (*P* < 0.01).

The effect of the fat-1 gene on CRC cell proliferation was verified by the expression of PCNA protein and transcript in the four mouse models (Fig 1). Colonic PCNA-positive cells and PCNA transcript were increased in obese diabetic mice 11 and 19 weeks following first DMH administration, implying crypt expansion, while remaining low in fat-1 mice (Fig 1). Most importantly, PCNA transcript and the number of PCNA-positive cells were significantly decreased in fxB mice as compared with the diabetic BKS.Cg db/db mice, implying suppression of DMH-induced diabetes-promoted CRC cell proliferation by (n-3) PUFA.

**Figure 2:**
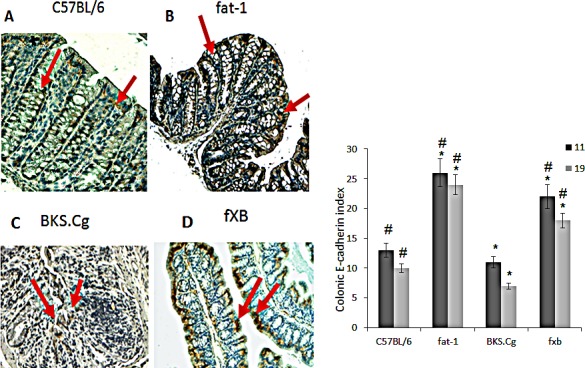
Colonic E-cadherin expression A-D. Representative colonic E-cadherin immunohistograms (magnification x 200) of C57BL/6 (A), fat-1 (B), BKS.Cg (C) and fXB (D) mice, following 11 weeks from first DMH injection. Arrows: E-cadherin positive nuclear staining. E. Colonic E-cadherin index 11 and 19 weeks from first DMH injection. Mean ± S.E. (n = 6 for each strain and time period). *Significant as compared with respective C57BL/6 control mice (*P* < 0.02). ^#^Significant as compared with respective BKS.Cg mice (*P* < 0.01).

Colonic E-cadherin was down-regulated in obese diabetic BKS.Cg db/db mice following 11 weeks of first DMH injection, while being highly expressed in fat-1 mice (Fig 2), implying epithelial differentiation. Introducing the fat-1 gene in the diabetic context resulted in high E-cadherin expression (Fig 2), implying high differentiation status. Similar results were observed following 19 weeks of first DMH administration (not shown).

### Expression of colonic HNF-4α, β-catenin and related transcripts

**Figure 3:**
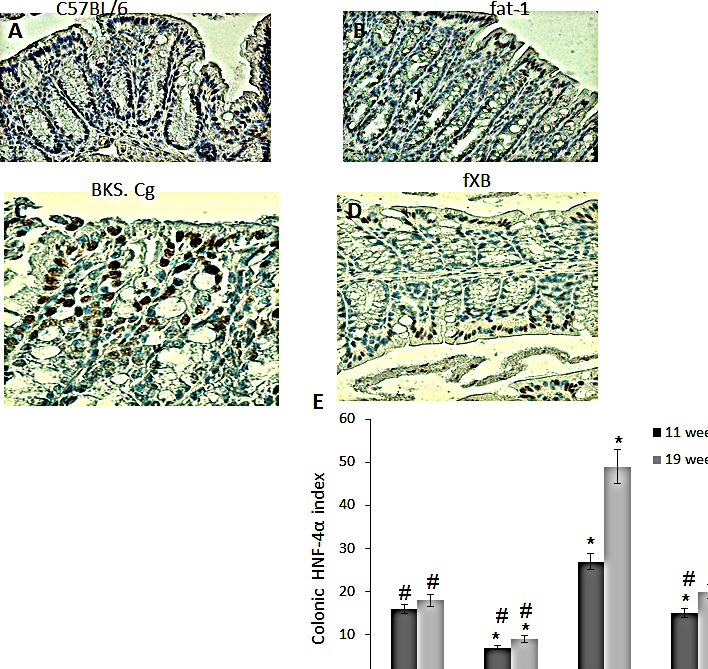
Colonic HNF-4α expression A-D. Representative colonic HNF-4α immunohistograms (magnification x 200) of C57BL/6 (A), fat-1 (B), BKS.Cg (C) and fxB (D) mice, following 11 weeks from first DMH injection. E. Colonic HNF-4α index 11 and 19 weeks from first DMH injection. Mean ± S.E. (n = 6 for each strain and time period). *Significant as compared with respective C57BL/6 control mice (*P* < 0.01). ^#^Significant as compared with respective BKS.Cg mice (*P* < 0.01).

Colonic HNF-4α expression in the four experimental mouse groups was evaluated following 11 and 19 weeks of first DMH administration (Fig 3). Colonic HNF-4α positive cells were increased in obese diabetic BKS.Cg db/db mice as compared with C57BL control mice, while being robustly decreased in fat-1 mice, both on 11 weeks and 19 weeks (Fig 3E) following first DMH administration. In line with that, the number of HNF-4α positive cells in BKS.Cg db/db mice has doubled during cancer development from week 11 (pre-cancer stage) to week 19, while remaining essentially constant in fat-1 mice (Fig 3E). Most importantly, the number of HNF-4α positive cells following 11 weeks of first DMH administration was significantly decreased in fxB mice as compared with obese diabetic mice, being further maintained at the C57BL control level on 19 weeks following first DMH administration.

**Figure 4:**
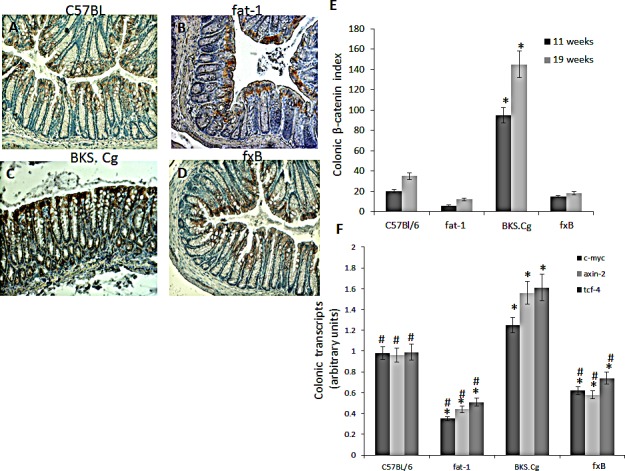
Colonic β-catenin expression A-D. Representative colonic β-catenin immunohistograms (magnifications x 200) of C57BL/6 (A), fat-1 (B), BKS.Cg (C) and fxB (D) mice, following 11 weeks from first DMH injection. E. Colonic β-catenin index 11 and 19 weeks from first DMH injection. Mean ± S.E. (n = 6 for each strain and time period). *Significant as compared with respective C57BL/6 control mice (P < 0.001). ^#^Significant as compared with respective BKS.Cg mice (*P* <0.001). F. Colonic β-catenin-responsive transcripts tcf-4, axin-2, and c-myc following 11 weeks from first DMH administration. Mean ± S.E. (n = 10 for each mice strain). *Significant as compared with respective C57BL/6 control mice (*P* < 0.001). ^#^Significant as compared with respective BKS.Cg mice (*P* < 0.01).

Colonic β-catenin expression and β-catenin/Tcf-responsive transcripts in the four mouse models was evaluated following 11 (Fig 4) and 19 weeks (not shown) of first DMH administration. CRC progression in obese diabetic mice was associated with a pronounced increase in colonic nuclear β-catenin. Introducing the fat-1 gene into the obese diabetic context resulted in abrogating nuclear β-catenin expression (Fig 4E). In line with that, introducing the fat-1 gene into the obese diabetic context resulted in suppressing β-catenin/Tcf-responsive transcripts (e.g., axin-2, tcf-4 and c-myc) (Fig 4F), implying decrease in β-catenin/Tcf-induced CRC progression.

### Suppression of proliferation-associated markers in CRC cell lines by (n-3) PUFA

**Figure 5:**
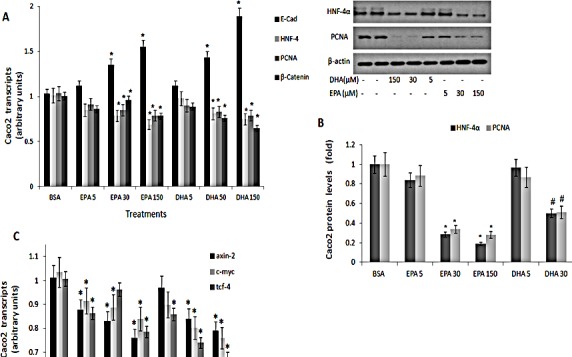
PCNA, E-cadherin, HNF-4α and β-catenin expression in Caco 2 cells treated with (n-3) PUFA Caco2 cells were treated with vehicle (BSA), eicosapentaenoic (EPA) or docosahexaenoic (DHA) (n-3) PUFA (μM) as indicated. A. PCNA, E-cadherin, HNF-4α and β-catenin transcripts were determined by qRTPCR. Respective transcript levels in the presence of BSA are defined as 1.0. Mean ± SE (n=6). *Significant as compared to the respective control (*P*< 0.004). B. HNF-4α and PCNA protein levels were determined by SDS-PAGE analysis, normalized to β-actin. Respective intensities in the presence of BSA are defined as 1.0. Mean ± SE (n=6). *Significant as compared to the respective control (*P*< 0.004 ). Inset- representative blots. C. β-catenin-responsive transcripts axin-2, tcf-4 and c-myc expression were determined by qRTPCR. Respective transcript levels in the presence of BSA are defined as 1.0. Mean ± SE (n=6). *Significant as compared to the respective control (*P*< 0.004).

(N-3) PUFA efficacy in suppressing proliferation-associated markers of CRC was further verified in Caco2 CRC cells. Specificity to (n-3) PUFA was evaluated by comparing (n-3) PUFA to (n-6) PUFA. Similarly to fat-1 mice, protein and transcript levels of HNF-4α, β-catenin and PCNA were dose dependently suppressed by EPA and DHA (Figs 5A,B), resulting in decrease in the β-catenin/Tcf-responsive transcripts axin-2, tcf-4 and c-myc (Fig 5C). Treatment with (n-3) PUFA further resulted in increase in E-cadherin (Fig 5A).

**Figure 6:**
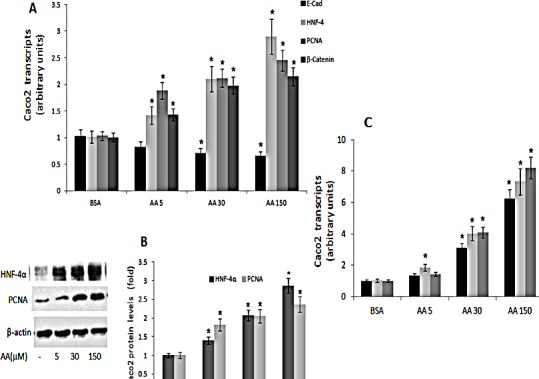
PCNA, E-cadherin, HNF-4α and β-catenin expression in Caco 2 cells treated with (n-6) PUFA Caco2 cells were treated with vehicle (BSA), or with arachidonic acid (AA) (n-6) PUFA (μM) as indicated. A. PCNA, E-cadherin, HNF-4α and β-catenin transcripts were determined by qRTPCR. Respective transcript levels in the presence of BSA are defined as 1.0. Mean ± S.E. (n=6). *Significant as compared with BSA (*P <* 0.004). B. HNF-4α and PCNA protein levels were determined by SDS-PAGE analysis, normalized to β-actin. Respective intensities in the presence of BSA are defined as 1.0. Mean ± SE (n=6). *Significant as compared to the respective control (*P*< 0.004 ). Inset- representative blots. C. β-catenin-responsive transcripts axin-2, tcf-4 and c-myc expression were determined by qRTPCR. Respective transcript levels in the presence of BSA are defined as 1.0. Mean ± SE (n=6). *Significant as compared to the respective control (*P*< 0.004).

In contrast, treatment of Caco2 cells with the (n-6) arachidonic acid resulted in dose-dependent decrease in E-Cadherin, with increase in HNF-4α, PCNA and β-catenin expression (Figs 6 AB), being accompanied by increase in axin-2, tcf-4 and c-myc transcripts (Fig 6C).

### MEDICA suppression of T2D-promoted colon cancer

MEDICA analogs have previously been reported to ameliorate T2D in animal models [16-19], and to mimic (n-3) PUFA activity in suppressing HNF-4α transcriptional activity [15, 20], prompting our interest in probing their efficacy in preventing the development of diabetes-promoted CRC, as compared with fat-1.

**Figure 7:**
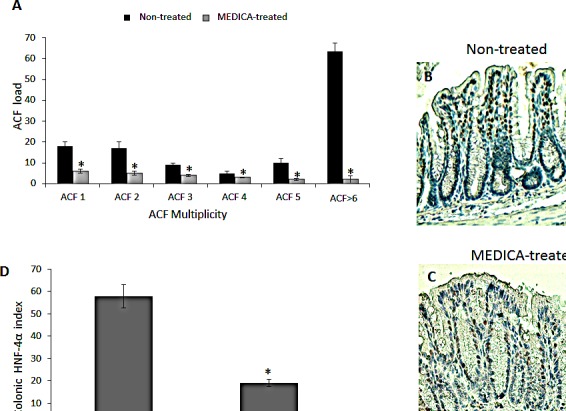
Suppression of diabetes-promoted CRC by MEDICA DMH-treated BKS.Cg mice were fed regular (non-treated) or MEDICA feed for 19 weeks following first DMH injection as described in Methods. A. ACF score. Mean ± S.E. (n = 10 in each group). *Significant as compared with mice fed regular feed (non-treated) (*P* < 0.05). B,C. Representative colonic HNF-4α immunohistograms (magnification x 200) of non-treated and MEDICA-treated mice. D. Colonic HNF-4α index. Mean ± S.E. (n = 10 per group). *Significant as compared with non-treated mice (*P* < 0.05).

**Figure 8:**
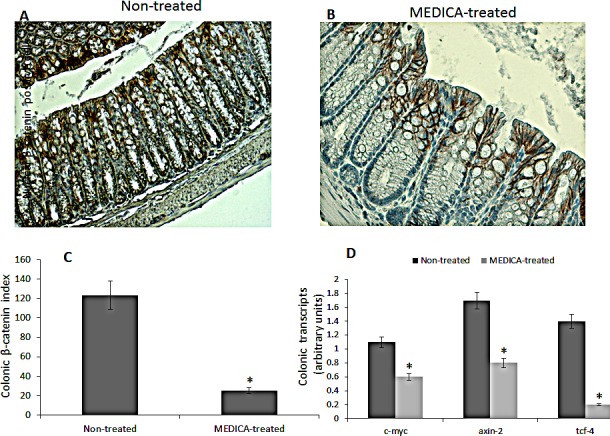
Effect of MEDICA on colonic β-catenin expression and β-catenin-responsive transcripts in diabetes-promoted CRC DMH-treated BKS.Cg mice were fed regular (non-treated) or MEDICA feed for 19 weeks following first DMH injection as described in Methods. A, B Representative colonic β-catenin immunohistograms (magnifications x 200) of non-treated and MEDICA-treated mice. C. Colonic β-catenin index. Mean ± S.E. (n = 10 per group) *Significant as compared with non-treated mice (*P* < 0.01). D. Colonic β-catenin-responsive transcripts tcf-4, axin-2, and c-myc. Mean ± S.E. (n = 10 per group). *Significant as compared with non-treated mice (*P* < 0.01).

MEDICA treatment of BKS.Cg db/db mice has been initiated one week following first DMH administration, and lasted for 19 weeks. MEDICA treatment resulted in decrease in body weight gain, accompanied by decrease in fasting plasma glucose, insulin and leptin ( Table 1), while maintaining food consumption (not shown). MEDICA treatment resulted in robust decrease in colonic ACF multiplicity, whereby ACF containing ≥ 6 foci became essentially absent by MEDICA (Fig 7A). That is in contrast to non-treated BKS.Cg db/db mice, being inflicted by high adenocarcinoma scoring. Decrease in ACF multiplicity by MEDICA has been further reflected by suppressing the expression of colonic HNF-4α (Fig 7B-D) as well as β-catenin and β-catenin/Tcf-responsive genes (Fig 8).

## DISCUSSION

Prevention and treatment of CRC by (n-3) PUFA has previously been studied in the context of sporadic CRC [10]. However, CRC in the obesity / T2D context involves higher morbidity and mortality risk [22, 23], due to induced increase in plasma insulin, IGF-1 and adipo/cytokines that promote cell survival [24], being further increased by treatment with insulin or β-cell secretagogues [25]. Indeed, in contrast to liver, muscle and adipose tissue where diabetes-induced increase in plasma insulin is compensated by diabetic insulin resistance, insulin-sensitive cancer cells in the diabetes context are exposed to high insulin and IGF-1 acting as growth factors. High risk of CRC promotion by diabetes has indeed been indicated by our previous study whereby colonic ontogenesis in db/db mice displayed CRC-like features with increase in proliferation and de-differentiation of epithelial colonocytes and goblet cells, being driven by increase in the expression and transcriptional activities of colonic HNF-4α and β-catenin/Tcf [13]. Hence, diabetes-promoted CRC presents a treatment challenge that surpasses that of sporadic CRC.

Carcinogen-induced CRC is shown here to be promoted by the diabetes context, in terms of increased ACF load, cell proliferation, and epithelial de-differentiation as verified by decrease in E-cadherin content. Most importantly, introducing the fat-1 gene into the diabetes context resulted in abrogating CRC development and cell proliferation, while suppressing the expression of HNF-4α, β-catenin and β-catenin/Tcf-responsive genes, and promoting epithelial differentiation. The specific activity of (n-3) PUFA in abrogating CRC, as contrasted with CRC promotion by (n-6) PUFA, was further verified here by testing the two PUFA back to back in CRC cell lines. Of note, profiles of cell proliferation, differentiation and the expression and activity of HNF-4α and β-catenin reported here for carcinogen-induced CRC in db/db, fat-1 and fXB mice are similar to those previously reported for colonic ontogenesis of respective mice [13], implying that HNF-4α and β-catenin may act as colonic ontogenic and oncogenic drivers in the diabetes context.

Abrogating CRC development by (n-3) PUFA may be ascribed to both, (n-3) PUFA efficacy in ameliorating the diabetic phenotype, combined with (n-3) PUFA efficacy in suppressing the expression and transcriptional activities of colonic HNF-4α and β-catenin/Tcf. The efficacy of (n-3) PUFA in suppressing CRC in respective cell lines may indeed point to their activity in suppressing CRC development independently of ameliorating the diabetic phenotype. The role played by HNF-4α in driving colonic oncogenesis conforms with previously reported findings whereby a. HNF-4α transcript and protein levels were increased by 2-3 folds in human CRC surgical samples when compared with their paired margin resections [26]; b. Silencing HNF-4α expression of HT29 CRC cells by SiHNF-4α resulted in suppressing their proliferation and in activating E-Cadherin expression [27]; c. Postnatal conditional deletion of HNF-4α resulted in robust decrease in polyp multiplicity in Apc^Min^ mice [26].

(N-3) PUFA efficacy in ameliorating the diabetic phenotype in obese diabetic animal models has previously been verified by partly replacing (up to 15%) dietary fat calories with (n-3) PUFA [28-30]. (N-3) PUFA feeding resulted in sensitization to insulin in skeletal muscle, liver and adipose tissue, with moderate decrease in fasting blood glucose and insulin. In line with that, fat-1 mice fed with high fat diet were partly protected from peripheral insulin resistance and glucose intolerance, being ascribed to inefficient biosynthesis of resolution mediators (e.g., Resolvin E1, Protectin D1) in liver and muscle [31]. Our present report extends and amplifies these previous studies by pointing out that high enough (n-3) PUFA may revert the db/db diabetic phenotype to the wild type profile. The potent anti-diabetic activity of the fat-1-generated PUFA, as compared with dietary (n-3) PUFA supplementation, may indicate a requirement for high (n-3) PUFA availability. Requirement for high-dose PUFA may account for the reported inefficacy of dietary (n-3) PUFA in controlling hyperglycemia in Western diabetic patients, while showing some efficacy in preventing diabetes in Asiatic subjects [32-34]. The requirement for high (n-3) PUFA availability may perhaps reflect its antidiabetic mode-of-action being mediated by activating AMPK [35].

HNF-4α has previously been reported by us to serve as target for long-chain fatty acids (LCFA) and their CoA-thioesters [15]. Thus, saturated LCFA-CoA activate and (n-3) PUFA suppress HNF-4α transcriptional activity, while (n-6) PUFA is neutral [15]. LCFA effects in modulating HNF-4α activity were accounted for by binding of respective acyl-CoAs to the acyl-CoA binding site of HNF-4α, followed by intramolecular hydrolysis of the CoA-thioester by HNF-4α thioesterase, with concomitant intramolecular channeling of the free acid into the free acid binding site of HNF-4α [20]. In line with that, CRC-like colonic ontogenesis in obese diabetic db/db mice, driven by increase in the transcriptional activity of colonic HNF-4α, was accompanied by robust increase in HNF-4α-bound (n-6) PUFA at the expense of decrease in HNF-4α-bound (n-3) PUFA [13]. Also, colonic ontogenesis in the fat-1 transgene or the fXB cross, driven by decrease in the transcriptional activity of colonic HNF-4α, was accompanied by increase in HNF-4α-bound endogenous (n-3) PUFA at the expense of decrease in HNF-4α-bound (n-6) PUFA [13]. Suppression of HNF-4α transcriptional activity by (n-3) PUFA may further be complemented by suppressing HNF-4α expression by (n-3) PUFA-activated AMPK [35-37]. The mode of action of (n-3) PUFA in suppressing colonic β-catenin expression and transcriptional activity still remains to be investigated.

Suppression of HNF-4α transcriptional activity by (n-3) PUFA requires the PUFA free acid or the PUFA-CoenzymeA (CoA) thioester, rather than PUFA esterified into endogenous triglycerides / phospholipids [15, 20]. Hence, treatment modes exploiting the anti-CRC efficacy of (n-3) PUFA may require relatively high doses of (n-3) PUFA, due to the rapid elimination of the PUFA free acid by either esterification into endogenous triglycerides / phospholipids or by beta-oxidation. However, fish consumption may provide only moderate (n-3) PUFA, while fish oil supplements or purified EPA / DHA (recommended at daily intake of up to 4 gr) may increase the endogenous (n-3) PUFA free acid to some extent only. Hence, it is doubtful whether fat-1-generated (n-3) PUFA content may be approached by dietary (n-3) PUFA supplementation. Hence, the management of CRC with concomitant diabetes calls for treatment strategies that suppress oncogenic drivers while maintaining glycemic control. Indeed, MEDICA analogs combine the two features of counteracting diabetes in animal models (e.g., Zucker, cp/cp, db/db, ob/ob) [16-19], while mimicking (n-3) PUFA in suppressing HNF-4α transcriptional activity [15, 20], and in activating AMPK [38]. In line with that, MEDICA analogs were previously reported to suppress proliferation and HNF-4α expression of cultured CRC cells, similarly to that of SiHNF-4α, and to suppress growth of CRC xenograft transplanted in immunocompromised mice [27].

The insulin-sensitizing anti-diabetic activities of MEDICA were indeed reproduced here in the CRC diabetic animal model, in terms of decrease in body weight gain, and fasting blood glucose, insulin and leptin, while maintaining food consumption. Also, in mimicking (n-3) PUFA efficacy, MEDICA treatment resulted in suppressing the expression and activity of colonic HNF-4α, β-catenin and β-catenin/Tcf-responsive genes. Most importantly, MEDICA treatment resulted in decreasing the ACF load, and in robustly abrogating the development of colorectal microadenoma of diabetes-promoted CRC. MEDICA efficacy in abrogating the diabetic phonotype and CRC development in the diabesity context was similar to that induced by the genetic fat-1 manipulation, implying its prospective therapeutic efficacy in the prevention and treatment of sporadic or diabetes-associated CRC.

## MATERIALS AND METHODS

### Materials

All chemicals and biochemicals were from Sigma Chemical Co. (St. Louis, MO), unless otherwise specified.

### Animals and experimental design

C57BL/6J and heterozygous BKS.Cg-+Lepr^db^/+Lepr^db^/OlaHsd (BKS.Cg db/db) mice were from Harlan laboratories, Ein Kerem, Jerusalem. Heterozygous BKS.Cg-Leprdb/Leprdb/OlaHsd (BKS.Cg) mice (Harlan) were bred to yield homozygous mice as verified by genotyping, their fasting blood glucose levels (Optimum Xceed/Plus; Abbot, UK), serum leptin (Quantikine Mouse Leptin Immunoassay kit; R & D Systems, Minneapolis, MN), and insulin levels (Mercodia ultrasensitive mouse insulin ELISA kit; Uppsala, Sweden). Heterozygous fat-1 mice were kindly provided by Jing X. Kang (Department of Medicine, Massachusetts General Hospital, and Harvard Medical Scholl, Boston, MA.), and were reproduced in our animal house. The presence of the fat-1 gene was confirmed by PCR genotyping as previously described [13]. The fat-1 phenotype was further verified by GC analysis of extracted fatty acids from the tail and colon as previously described [13]. fxB mice were produced by crossing the BKS.Cg db/db mice with fat-1 mice. First generation fXB mice were selected for the studies described, upon confirming their genotype and phenotype as previously described [13]. Animals were kept in plastic cages with wire tops in a light / temperature-controlled / Specific Pathogen Free (SPF) conditions, and maintained on Teklad 2018S standard rodent diet (54% carbohydrate, 18% fat, 24% protein energy). Where indicated, mice were fed with MEDICA analog {(CH2)_12_ –[C(CH3)_2_-COOH]_2_} mixed in powdered chow (0.05% (W/W). Colon cancer was induced according to Tirosh et al., 2005 [39]. Briefly, 2 month old mice [C57BL/6J, BKS.Cg db/db, fat-1 and fxB] were injected s.c. once a week, for 4 weeks, with 20 mg/kg body weight of 1,2-dimethylhydrazine (DMH, Fluka chemical Co., Buchs, Switzerland) in saline-1.5% EDTA (w/v) (pH 6.5). Mice were sacrificed by cervical dislocation 11 and 19 weeks from first DMH injection. Fasting blood glucose was measured using glucose strips (Optimum Xceed/Plus, Abbot). Serum leptin was measured using Quantikine Mouse Leptin Immunoassay kit. Serum insulin was measured using Mercodia ultrasensitive mouse insulin ELISA kit.

### Colon cancer cell line

Caco 2 (human CRC cell line) (ATCC, Manassas, VA) cells were cultured in 6-well plates at 37°C, 5% CO2 in DMEM supplemented with 10% (v/v) FCS, 2mM L-glutamine and 0.2% (v/v) penicillin-streptomycin. Upon reaching confluence, cells were treated twice every 24 h with 5, 30 and 150 μM EPA, DHA, or AA as indicated. Briefly, respective fatty acid stock solutions (100 mM) in 95% sterile ethanol were diluted in DMEM containing 0.4 % fatty acid-free bovine serum albumin (BSA), followed by 30 min incubation at 37ºC. Control treatment consisted of 0.2 % (v/v) ethanol and 0.4 % (w/v) BSA in DMEM.

### Aberrant crypt foci (ACF) scoring

Following cervical dislocation, 2 cm of distal colon was immediately removed and flushed with cold saline solution containing 2 mM DTT. Colons were dissected longitudinally, fixed flat between filter paper for 72 h in 4% buffered formaldehyde, were stained with Methylene Blue (0.1% in saline solution) for 10–15 min, and then washed with saline for 10 min. Colonic mucosal ACF were examined under a light microscope (Olympus, Tokyo, Japan) at a magnification of x20 / x40. ACF were characterized by their crypt diameters, slit-like opening, increased staining and size of pericryptal zone. ACF were scored and classified into 5 groups by their multiplicity. Six or more crypts / focus were scored as a separate group, being characterized by their highly enlarged crypts, slit-shaped lumina, and microadenoma formations. Overall Foci load was calculated as the number of foci in each ACF group along the colonic sample tested.

### ImmunoHistoChemistry (IHC)

Colon tissue samples were fixed overnight at 4 °C in 4% buffered formaldehyde, washed in phosphate buffered saline (PBS), dehydrated through up-grade series of ethanol dilutions, cleared in Histoclear (Kaltek, Padova, Italy) and finally embedded in paraffin. 5 μm thick sections were cut on Superfrost Plus microscope slides (D-38116, Menzel GmbH & Co KG, Braunschweig, Germany) and dried overnight at 40°C. Slides were de-waxed and rehydrated through down-grade series of ethanol dilutions to PBS containing 0.05 % Tween (PBST). Antigen retrieval was carried out by microwave heating for 1 min in citrate buffer (0.1 M, pH 6.5). The slides were then cooled, incubated with 3% (v/v) hydrogen peroxide solution in PBS, washed in PBST, and subjected to protein blocking for 20 min in PBS solution containing 5% normal horse serum and 1% normal goat serum (Biological Industries Israel Beit-Haemek, Kibbutz Beit Haemek, Israel). The slides were then incubated overnight with primary antibodies as indicated: anti-proliferating cell nuclear antigen (PCNA) (M0879, Dako Corp. Glostrup, Denmark) (used at 1:200 dilution); anti–E-cadherin (610181, BD Biosciences) (used at 1:100 dilution); monoclonal mouse anti-human HNF-4α (ab41898; Abcam, Cambridge, UK) (used at 1:200 dilution); and monoclonal mouse anti-human β-catenin (Santa Cruz Biotechnology, Dallas, TX) (used at 1:100 dilution). Following overnight incubation with primary antibodies the slides were washed with PBST, were further incubated with the protein blocking solution for 20 min., followed by incubating with respective secondary antibody: goat anti-mouse and goat anti-rabbit antibodies (Jackson Laboratory, Bar Harbor, ME) (used at 1:200 dilution). Following color development using DAB chromogen solution (Labvision, Fremont, CA), slides were counterstained with Gill No.3 Hematoxylin solution, rinsed with water and mounted with Fluoromount. Slides were examined with an inverted light microscope (Olympus) at x10 and x20 magnification. Colonic PCNA, E-cadherin, HNF-4α and β-catenin indices were calculated as the percentage of colonic respective stained area relative to total colonic area of 6 individual sections prepared from each animal, using ImageJ tools (National Institutes of Health, Bethesda, MD).

### Western blot analyses

Western blot analyses were performed as previously described [27]. Proteins were visualized using ECL kit (Amersham Biosciences, Buckinghamshire, UK).

### Real-Time PCR

Total RNA of tissue samples and colon cancer cells was extracted using TRI Reagent according to manufacturer instructions. RNA concentration and purity were validated by verifying the 260/280 and 230/280 ratios (Nanodrop ND-1000 V.3.1.0, NanoDrop Technologies Inc, Wilmington, DE). Following DNAse-treatment, 2 micrograms of RNA were reverse-transcribed using Superscript First-Strand cDNA kit, followed by subjecting the cDNA to quantitative real-time PCR using the SYBR Green Master kit and the ABI Prism 7300 Sequence Detection System (Applied BioSystems, Grand Island, NY). Primers were designed using Primer Express v.2.0 (Applied BioSystems) and their specificity was validated by respective dissociation curves. Primers were validated over four orders of magnitude and analyzed using 7300 System Software. Primers for mouse genes consisted of: PCNA [F: 5′- TTT GAG GCA CGC CTG ATC C-3′, R: 5′-GGA GAC GTG AGA CGA GTC CAT-3′]; c-myc [F: 5′-TGG ACA CGC TGA CGA AA GT-3′, R:5′-AGG CGA AGC AGC TCT ATT TCT-3′]; axin-2 [F: 5′-CCA TGA CGG ACA GTA GCG TA-3′, R: 5′-GC CAT TGG CCT TCA CAC T-3′]; tcf4 [F: 5′-GAA AAG TTC CTC CGG GTT TG-3′, R: 5′-GAG AGT TCC CTG GCT GTG TC-3′]; Mouse genes were normalized to mouse ribosomal protein S18 (RPS18) gene [F: 5′-GTA ACC CGT TGA ACC CCATT-3′, R: 5′-CCA TCC AAT CGG TAG TAG CG-3′]. Primers for human genes consisted of: axin2 [F:5′-GAG AGT GAG CGG CAG AGC-3′, R: 5′- CGG CTG ACT CGT TCT CCT-3′]; tcf 4 [F: 5′-CAA CGA ACA CAG CGA ATG TT-3′, R:5′- TTA GGA GCG CTC AGG TCT GT-3′]; c-myc [F: 5′- GAC AAG AGG CGG ACA CAC AA-3′,R:5′-GGA TGT AGG CGG CTT TT-3′]; HNF-4α [F:5′-TGT CCC GAC AGA TCA CCTC-3′, R: 5′-CAC TCA ACG AGA ACCA GCAG-3′]; PCNA [F:5′- AGG CAC TCA AGG ACC TCA TCA-3′, R:5′- GAG TCC ATG CTC TGC AGG TTT-3′], E-cadherin [F: 5′-GTG ACT GAT GCT GAT GCC CCC AAT ACC-3′; R: 5′-GAC GCA GAA TCA GAA TTA GGA AAG CAA G-3′], β-catenin [F: 5′-TGA GGA CAA GCC ACA AGA TTA C-3′; R: 5′-TCC ACC AGA GTG AAA AGA ACG-3′]. Human genes were normalized to human GAPDH [F: 5′- AGC CAC ATC GCT CAG ACA C-3′, R: 5′- GCC CAA TAC GAC CAA ATC C-3′]. Fold change in target gene expression was calculated by the comparative CT method also referred to as the 2-^ΔΔct^ method, (Applied BioSystems). Transcript values for respective mice groups represent mean ± S.E. of independent triplicates, whereby each biological triplicate was analyzed in technical triplicate.

### Statistical analysis

Data are presented as mean ± S.E., using t-test and one-way analysis of variance (ANOVA) using JMP 5.1 tools (Marlow, Buckinghamshire, UK).

## SUPPLEMENTARY FIGURE


