# Supplementary material for: Prevention of diabetes-promoted colorectal cancer by (n-3) polyunsaturated fatty acids and (n-3) PUFA mimetic
Source: Oncotarget. 2014 Sep 8;5(20):9851–63. doi: 10.18632/oncotarget.2453 (PMC4259442; doi:10.18632/oncotarget.2453)
Supplement: Supplementary file 1 [file oncotarget-05-9851-s001.pdf]

Prevention of diabetes-promoted colorectal cancer by (n-3) polyunsaturated fatty acids and (n-3) PUFA mimetic

Supplementary Material

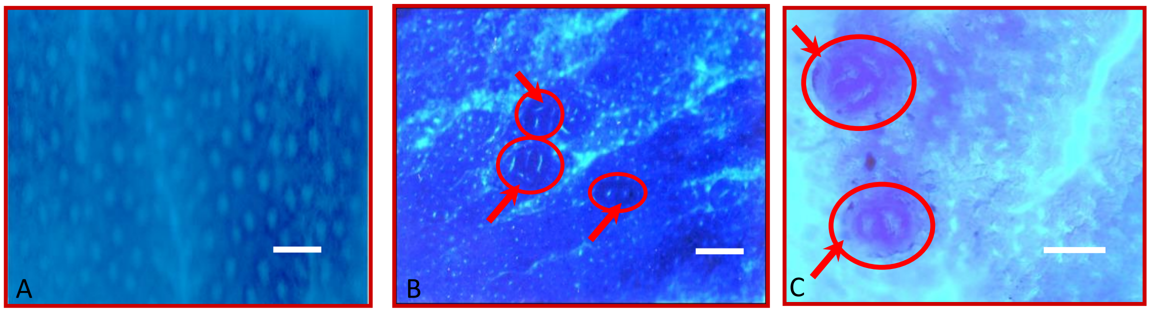

Figure S1: Representative sections of colonic mucosa stained with Methylene Blue. Bars =100µm. (A) Representative colon section of control non-carcinogen treated mice. (B) ACF of DMH-treated C57BL/6 mice. (C) ACF consisting of 2 and 3 foci shown at higher magnification.
